# Electrical stimulation enhances neuronal cell activity mediated by Schwann cell derived exosomes

**DOI:** 10.1038/s41598-019-41007-5

**Published:** 2019-03-12

**Authors:** Ming Hu, Li Hong, Cheng Liu, Shasha Hong, Songming He, Min Zhou, Guotao Huang, Qian Chen

**Affiliations:** Department of Gynecology and Obstetrics, Renmin Hospital of Wuhan University, 238 Jiefang Road, Wuhan, 430060 Hubei Province China

## Abstract

Electrical stimulation (ES) therapy has good effects in patients with nervous system injury-related diseases. ES promotes nerve cell regeneration and stimulates Schwann cells to express neurotrophic factors. The incidence of stress urinary incontinence (SUI) among elderly people is increasing. Some studies suggest that damage to the pudendal nerve is closely related to the pathogenesis of SUI. It has also been found that pelvic ES can reduce SUI symptoms in a rat model of SUI caused by pudendal nerve injury. Clinically, pelvic floor electrical stimulation is effective in patients with mild to moderate SUI. These studies indicate that ES may ameliorate damage to the pudendal nerve and thus achieve the goal of SUI treatment, although the mechanism of action of this treatment remains unclear. Therefore, the purpose of the present study was to clarify the relationships among ES, neural cells and Schwann cells at the cellular level. We applied ES to nerve cells at 100 mV/mm or 200 mV/mm for 0, 0.5, 1, or 2 h to investigate changes in nerve cell activity. We then co-cultured the nerve cells with Schwann cells to explore the influence of single-culture and co-culture conditions on the nerve cells. Compared to non-ES, ES of the nerve cells increased their activity. Compared to those in single culture, co-cultured nerve cells exhibited an additional increase in activity. We also found that Schwann cell derived exosomes could promote the activity of nerve cells, with glutamate and calcium ions playing a potential role in this process. These results suggest that the mutual regulation of neural cells and Schwann cells plays an important role in the process by which ES ameliorates neurological function, which may provide a basis for subsequent studies.

## Introduction

Electrical stimulation (ES) therapy plays an important role in delaying muscle atrophy in hemiplegic patients and promoting neuromuscular function recovery and has beneficial effects in patients with nervous system injury-related diseases^1–5^. Studies have confirmed that current stimulation within the safety limits activates the damaged neuromuscular system, promotes the electrical activity of neuronal cells and induces repair of synapses, thereby promoting the growth of nerve cells^6^. Current stimulation also slows neurological synaptic degradation and enhances myelin formation, and it might ultimately promote the regeneration of new nerve cells and their innervation of muscle cells^7^. In addition, studies have demonstrated that Schwann cells begin to highly express neurotrophic factors after ES, and these factors are then continuously released to the injured nerves, thus improving the nerve regeneration microenvironment, creating a good platform for nerve repair^8,9^, and promoting axonal regeneration.

Stress urinary incontinence (SUI) is a type of pelvic floor dysfunction, which presents as the spontaneous leakage of urine when abdominal pressure increases during the state of bladder detrusor relaxation^10^. Regarding aetiology, pudendal nerve injury is an important factor that leads to the occurrence of SUI^11^, which reduces the innervation of pelvic floor muscles. Studies have confirmed that SUI patients may exhibit pelvic floor muscle denervation through pelvic floor electromyography, nerve conduction velocity, pelvic floor muscle pathology and nerve fibre immunohistochemical staining^12–14^. In addition, animal experiments demonstrated that damaging the pudendal nerve of female rats can model postpartum SUI^15^, and the degree of damage to the pudendal nerve determines both the extent of pelvic floor function injury and the recovery time. Clinically, one physical treatment for SUI is pelvic electrical stimulation (PES), which shows good clinical effects for patients with mild or moderate symptoms^16–18^. Damaser^19^ used a rat model of pudendal nerve crush to confirm that ES of the pudendal nerve increases the expression of BDNF and βII-tubulin in Onuf’s nucleus and improves the symptoms of SUI caused by pudendal nerve crush. However, the internal mechanism by which ES therapeutically benefits SUI needs to be further explored.

Glutamate is the excitatory neurotransmitter in the nervous system. Cavus^20^ found that ES causes changes in the levels of glutamate release from hippocampal cells. In addition, Carsten^21^ confirmed that in the central nervous system, glutamate secreted by nerve cells can promote calcium influx in oligodendrocytes through binding to calcium-permeable ionotropic glutamate receptors on oligodendrocytes, thereby inducing the release of oligodendrocyte extracellular mass. The glial cells in the peripheral nervous system are called Schwann cells^22^. Exosomes are vesicle-like structures that are surrounded by a lipid bilayer and have a diameter of 40–150 nm. Studies have suggested that Schwann cell-derived exosomes play a role in promoting nerve regeneration and repair^23^. Therefore, we hypothesized that ES may repair pudendal nerve injury by increasing the activity of nerve cells via a process involving Schwann cell derived exosomes, thereby achieving the goal of treating SUI.

## Results

### ES increases dorsal root ganglion (DRG) cell viability, and the optimal parameters are 100 mV/mm for 1 h

**Figure 1:**
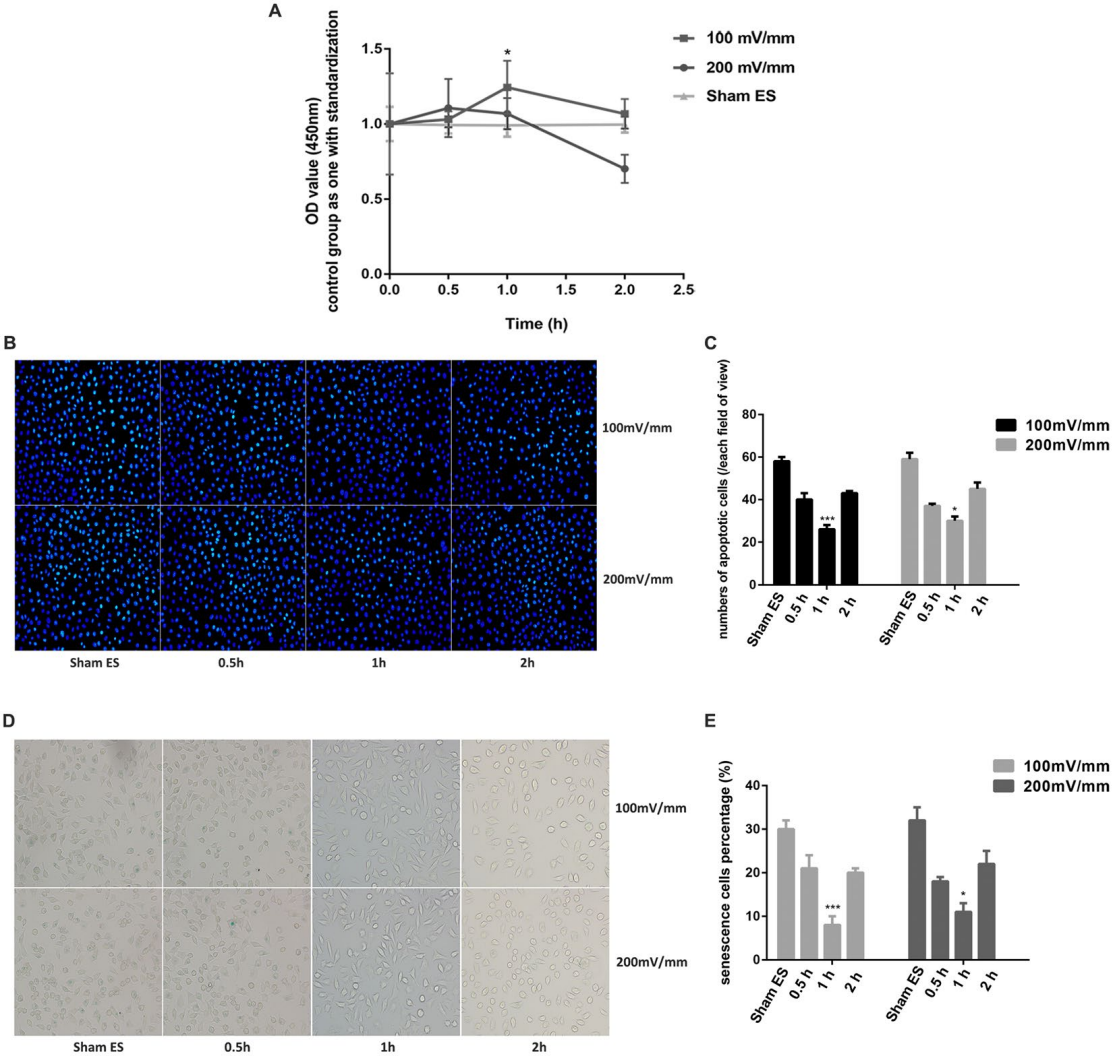
DRG activity after electrical stimulation. (**A)** DRG cell proliferation activity was measured using a CCK-8 kit and analysed using the optical density value, with the following electrical stimulation parameters: an electrical strength of 100 mV/mm or 200 mV/mm and a stimulation time of 0.5, 1, or 2 h. The Sham ES group was maintained under the same culture conditions as the electrical stimulation groups at each time point. The value of the Sham ES group was standardized to 1. **(B**,**C)** DRG cell apoptosis staining at a 200 × magnification. Blue fluorescence shows the nuclei of normal DRG cells, whereas the nuclei of apoptotic cells were partially or completely stained with a whitish fluorescence. **(D**,**E)** DRG cell senescence staining at a 200 × magnification. Deep blue cells viewed under the microscope represent senescent cells. Data are expressed as the mean ± SD. Each set of experiments was repeated 3 times. *p < 0.05, ***p < 0.01, compared to the non-electrical stimulation group.

To investigate the effects of ES under different conditions on DRG cells and to identify the optimal parameters with the most significant impact on DRG cells, we electrically stimulated DRG cells using the following ES parameters: an electrical strength of 100 mV/mm or 200 mV/mm and a stimulation time of 0.5, 1, or 2 h. The activity of DRG cells was measured after ES. As shown in Fig. 1, cell proliferation, as detected by Cell Counting Kit (CCK)-8, first increased and then decreased with increasing ES time (Fig. 1A). The optical density values following exposure to ES in the non-ES group (Sham ES), 100 mV/mm 0.5 h group, 100 mV/mm 1 h group, 100 mV/mm 2 h group, 200 mV/mm 0.5 h group, 200 mV/mm 1 h group and 200 mV/mm 2 h group were 1.000 ± 0.337, 1.031 ± 0.053, 1.244 ± 0.178, 1.067 ± 0.099, 1.106 ± 0.194, 1.068 ± 0.104, and 1.701 ± 0.094, respectively, with the Sham ES group used for standardization. The optical density values at each time point (0 h, 0.5 h, 1 h, and 2 h) for the Sham ES group were 1.000 ± 0.114, 0.993 ± 0.057, 0.991 ± 0.075, and 0.996 ± 0.053, respectively, with the 0 h group used for standardization, and there were no significant differences among groups (p > 0.05). Using the parameters of 100 mV/mm and 1 h, the activity of DRG cells was significantly increased compared to that of the other groups, and this difference was statistically significant (p < 0.05). When the ES time was increased to 100 mV/mm for 2 h, cell activity began to decline, and this value was not significantly different from that in the control group (p > 0.05). When the electrical strength was 200 mV/mm, the cell viability of DRG cells was slightly higher than that at 100 mV/mm with a stimulation time of 0.5 h (p > 0.05), but there were no statistically significant differences. Additionally, the cell activity at 200 mV/mm was lower than that at 100 mV/mm when the stimulation time was 1 h (p < 0.05).

We used a Hoechst staining kit to detect the occurrence of apoptosis after ES of DRG cells. After treatment, the DRG cells showed a normal blue nucleus under the fluorescence microscope if the cells were normal and healthy, whereas the nuclei of apoptotic cells were partially or completely stained with a whitish colour. The incidence of apoptotic cells after ES is shown in Fig. 1C. DRG cell apoptosis first decreased and then increased with increasing ES time (Fig. 1B,C). Apoptosis was lowest at 100 mV/mm after 1 h of exposure, which was significantly different from that of the other groups (p < 0.05). We also used a β-galactosidase staining kit to measure DRG cell senescence. Deep blue cells viewed under the microscope represented senescent cells (Fig. 1D). According to the histogram, with increasing ES time, the occurrence of cell senescence showed a tendency to initially decline and then increase, with a pattern similar to that observed for cell apoptosis (Fig. 1E). The lowest incidence of cell senescence was measured at 100 mV/mm after 1 h of exposure, and this difference was statistically significant (p < 0.05). There were no significant differences in cell apoptosis or cell senescence at any time point (0 h, 0.5 h, 1 h, or 2 h) among the Sham ES groups (p > 0.05) (Supplementary Fig. 1).

### ES increases the release of glutamate from DRG cells

**Figure 2:**
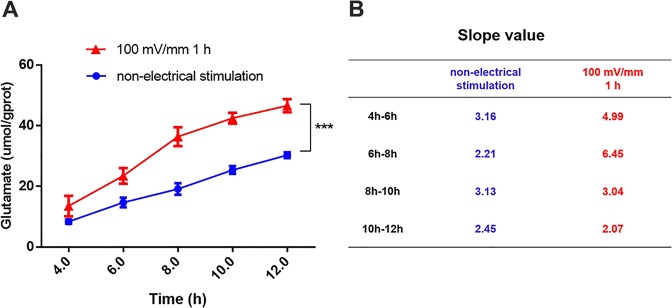
Detection of glutamate secretion after electrical stimulation. (**A)** The red line shows the glutamate secretion curve after electrical stimulation at 100 mV/mm for 1 h, and the blue line represents the non-electrical stimulation group. **(B)** The slope of the secretory curve for each time period for the 100 mV/mm 1 h groups and the non-electrical stimulation groups. Data are expressed as the mean ± SD. Each set of experiments was repeated 3 times. ***p < 0.01, compared to the non-electrical stimulation group.

To investigate the effect of ES on the secretion of glutamate by DRG cells, we cultured DRG cells after ES (fixed at 100 mV/mm for 1 h based on Fig. 1) uniformly in a 6-well plate at a concentration of 7–8*10^5^ cells/ml. Cell supernatants were then collected at 2, 4, 6, 8, 10, and 12 h, and glutamate levels in the supernatant were measured. Because there was a large number of non-adherent cells in the 6-well plate, we abandoned the 2 h group. We used non-electrically stimulated DRG cells as a control group, called the non-ES group, and adjusted the cell concentration to 7–8*10^5^ cells/ml. The cells from this group were also placed in an incubator for ES. After ES of the experimental groups, the cells were digested at the same time as the experimental group and then transferred to a 6-well plate. As seen in Fig. 2, we found that ES induced an increase in glutamate secretion by DRG cells. In supernatants collected from the ES group at 4, 6, 8, 10, and 12 h, the concentrations of glutamate were 13.50 ± 3.40, 23.47 ± 2.58, 36.38 ± 3.11, 42.45 ± 1.77 and 46.58 ± 2.15 μmol/g protein, respectively. The glutamate concentrations in the non-ES group supernatants at 4, 6, 8, 10, and 12 h were 8.38 ± 0.71, 14.70 ± 1.60, 19.13 ± 1.89, 25.39 ± 1.29 and 30.29 ± 0.95 μmol/g protein, respectively. The glutamate concentrations in the ES group at each time point were all higher than those in the non-ES group, and these differences were statistically significant (p < 0.05). In a curve of glutamic acid secretion at 100 mV/mm for 1 h, the slopes at 4–6 h, 6–8 h, 8–10 h, and 10–12 h were 4.99, 6.45, 3.04, and 2.07, respectively (Fig. 2B). The peak of glutamate secretion from DRG cells occurred at 6–8 h after ES, and the secretion of glutamate began to decrease after 8 h.

### Glutamate can promote the secretion of Schwann cell derived exosomes, and Ca^2+^ is involved in this process

**Figure 3:**
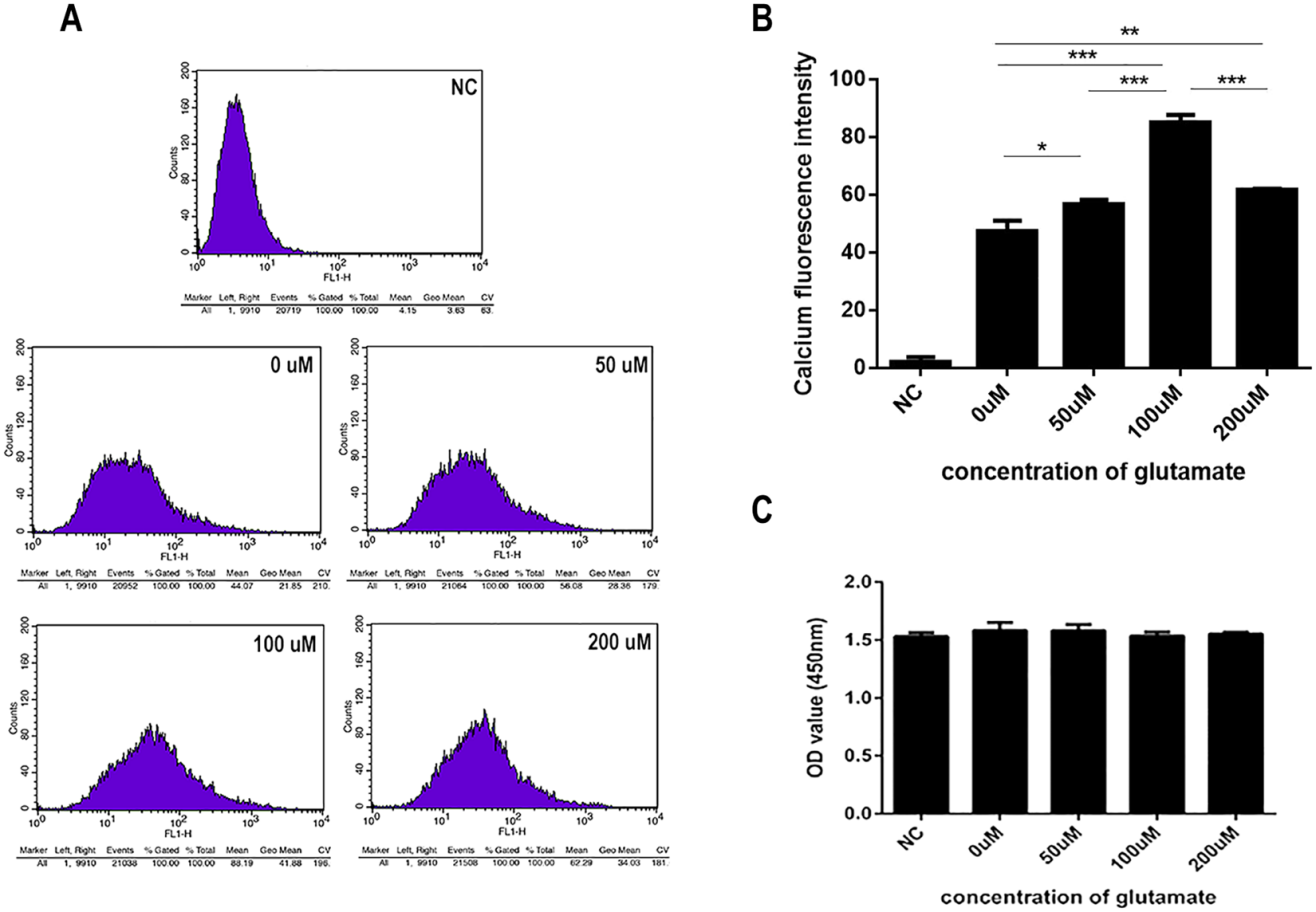
Effects of exogenous glutamate on intracellular calcium concentrations in Schwann cells. (**A**,**B)** Different concentrations of glutamate affect the calcium concentration in Schwann cells. B shows the quantification of the calcium fluorescence intensity. **(C)** Changes in the proliferative activity of Schwann cells after administration of different glutamate concentrations for 5 h. Data are expressed as the mean ± SD. Each set of experiments was repeated 3 times. *p < 0.05, ***p < 0.01, compared to the 0 μM group.

**Figure 4:**
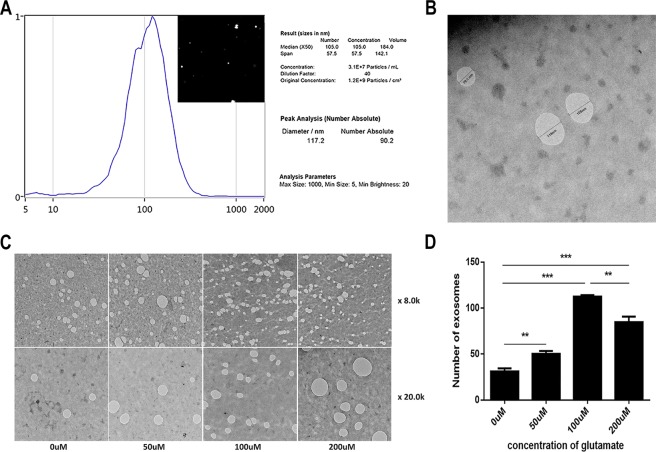
Effects of exogenous glutamate on exosome secretion by Schwann cells. (**A**,**B)** NanoSight and electron microscopy were used to determine whether exosomes were successfully extracted. The size of most exosomes was approximately 117 nm. **(C**,**D)** Changes in exosome secretion after treatment with different concentrations of glutamate at 8,000 × and 20,000 × magnification. The white vacuole-like structure in C represents exosomes. Data are expressed as the mean ± SD. Each set of experiments was repeated 3 times. **p < 0.05, ***p < 0.01, compared to the 0 μM group.

To determine whether glutamate can influence the secretion of exosomes by Schwann cells (RSC96), we administered exogenous glutamate at 0, 50, 100, or 200 μmol/L to Schwann cells for 5 h based on published studies^21^ and then measured the effects of glutamate on exosome secretion and intracellular calcium ion concentrations. To ensure that high-concentration and long-term glutamate stimulation was not toxic to the Schwann cells, we measured cell viability at 200 μmol/L for 5 h, confirming that the activity of Schwann cells was not significantly changed under these conditions (på 0.05) (Fig. 3C). After treating the Schwann cells with glutamate, we collected the supernatant and extracted the exosomes. We confirmed that exosomes were successfully extracted using NanoSight and electron microscopy (Fig. 4A,B). Electron microscopy showed that the number of exosomes secreted by Schwann cells increased with increasing glutamate concentrations. However, when the concentration reached 200 μmol/L, exosome secretion began to decrease slightly (Fig. 4C,D).

Changes in intracellular calcium levels affect the exocytosis of cells. To explore whether calcium ions play a role in the secretion of exosomes by Schwann cells, we measured changes in the intracellular calcium concentration after glutamate intervention (Fig. 3A,B). The results showed that the intracellular calcium concentration of Schwann cells reached its highest value at 100 μmol/L, which is consistent with the change in exosome secretion. This result may indicate that calcium ions play an important role in the secretion of exosomes by Schwann cells after stimulation with glutamate.

### Culturing DRG cells using supernatant from glutamate-treated Schwann cells can increase the activity of nerve cells

**Figure 5:**
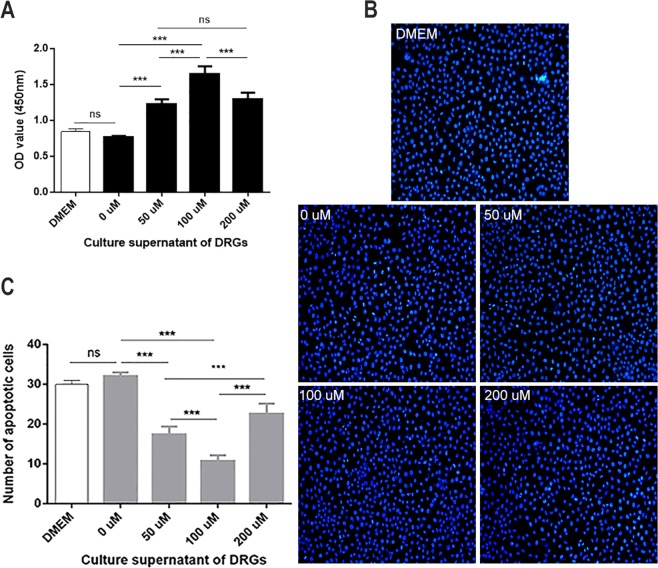
Effect of supernatant from glutamate-treated Schwann cells on DRGs. (**A)** Changes in the proliferation of DRGs after treatment with different concentrations of supernatants. **(B**,**C)** Apoptotic staining of DRGs after treatment with different concentrations of supernatants at a magnification of 200x. Blue fluorescence indicates the nuclei of normal DRG cells, whereas the nuclei of apoptotic cells were partially or completely stained with a whitish fluorescence. Data are expressed as the mean ± SD. Each set of experiments was repeated 3 times. *p < 0.05, ***p < 0.01, compared to the negative control group. *p < 0.05, ***p < 0.01, compared to the 0 μM group.

Since glutamate can stimulate Schwann cells to secrete exosomes, we used supernatants from glutamate-treated Schwann cells to culture DRG cells to determine whether these supernatants would affect DRG cell activity. Similarly, we examined cell viability and apoptosis. As shown in Fig. 5, the proliferative activity of nerve cells increased with increasing glutamate concentrations (Fig. 5A). The incidence of apoptosis gradually decreased at the same time (Fig. 5B,C). When the concentration of glutamate was 100 μmol/L, the activity of DRG cells was highest, with statistically significant differences compared to the first three groups (p < 0.01). After the concentration increased to 200 μmol/L, DRG cell activity began to show a decreasing trend, with a significant difference compared to the activity in the 100 μmol/L group (p < 0.01).

### DRG cells co-cultured with Schwann cells exhibit greater nerve cell activity than those cultured alone after ES, and this process is related to Schwann cell-derived exosomes

**Figure 6:**
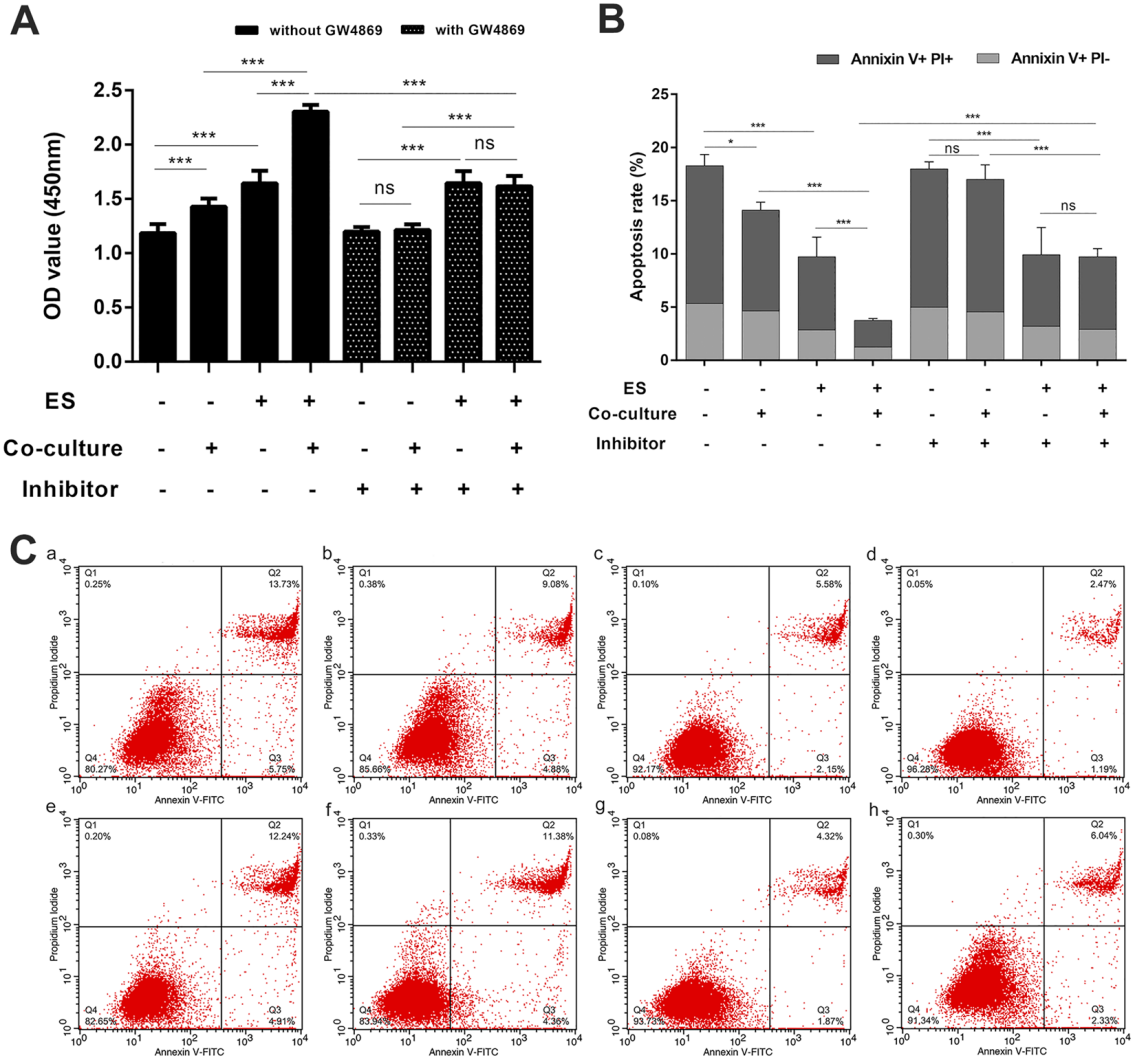
Effects of co-culture on DRG cell activity after electrical stimulation. (**A)** Changes in DRG cell proliferation activity under different treatment conditions. **(B)** Quantitative changes in the apoptosis of DRG cells under different treatment conditions. **(C)** Detection of DRG cell apoptosis under different treatment conditions. (a) without electrical stimulation, without co-culture, without inhibitor group; (b) without electrical stimulation, with co-culture, without inhibitor group; (c) with electrical stimulation, without co-culture, without inhibitor group; (d) with electrical stimulation, with co-culture, without inhibitor group; (e) without electrical stimulation, without co-culture, with inhibitor group; (f) without electrical stimulation, with co-culture, with inhibitor group; (g) with electrical stimulation, without co-culture, with inhibitor group; and (h) with electrical stimulation, with co-culture, with inhibitor group. Data are expressed as the mean ± SD. Each set of experiments was repeated 3 times. *p < 0.05, ***p < 0.01.

Based on the above results, we co-cultured DRG cells with Schwann cells after ES and observed the changes in DRG cells. At the same time, we pretreated Schwann cells with the exosome secretion inhibitor GW4869 and then co-cultured Schwann cells and DRG cells to detect the changes in DRG cells and the role of exosomes in this process. We used CCK-8 staining to detect the proliferation of DRG cells and Annexin V/PI double-staining with flow cytometric analysis to measure cell apoptosis. As shown in Fig. 6, in groups without GW4869 treatment, the proliferation of DRG cells after ES was increased (Fig. 6A), and the apoptotic ratio was decreased (Fig. 6B,C) compared to that in DRG groups that received non-ES (p < 0.01). At the same time, the proliferation of DRG cells after co-culture was increased, and the apoptotic ratio was decreased compared to that in non-co-cultured DRG groups (p < 0.05). In the presence of GW4869, the proliferation of DRG cells after ES was increased, and the apoptotic ratio was decreased compared to that in DRG groups that received non-ES (p < 0.01). However, there was no significant difference in proliferation or the apoptotic ratio between the co-cultured groups (p > 0.05). In addition, under the conditions of co-culture and ES, adding GW4869 significantly reduced the activity of the cells and increased the rate of apoptosis (p < 0.01).

## Discussion

Starting in 1976, Wilson^24^ *et al*. performed a large number of animal experiments and confirmed that ES significantly accelerates the regeneration of damaged peripheral nerves. Research on ES and peripheral nerve regeneration has gradually been increasing, with most studies supporting the idea that ES is a treatment that promotes nerve regeneration and accelerates the recovery of nerve function^25–27^. However, the mechanism by which ES promotes peripheral nerve regeneration remains unclear. The pudendal nerve, an important peripheral nerve, dominates all striated muscles of the pelvic floor, including the urethra and anal sphincter, and it plays an extremely important role in the maintenance of pelvic floor function^22,28^. Pudendal nerve injury is an important factor in the occurrence of SUI, and the degree of nerve injury determines the recovery of SUI. Clinical studies have shown that pudendal nerve stimulation has good effects for patients with bladder dysfunction^29^. In a preliminary study of this concept, C57BL/6 mice underwent vaginal distension (VD) to serve as models of SUI, in which ES was shown to ameliorate symptoms^30^. In addition, frequent ES improves pudendal nerve recovery in a dual simulated childbirth injury model^31^. These findings indicate that ES plays a role in the treatment of SUI by stimulating damaged pudendal nerves, with an unclear mechanism.

This study mainly focused on the cellular level, with the aim of exploring the mechanisms underlying the pudendal nerve pro-healing process after ES. In this study, primary culture of rat pudendal neuronal cells was originally planned, but this approach failed after repeated attempts. We reviewed the literature and found that most of the studies on peripheral nerve injury were performed with DRG cells^32–34^. Therefore, we chose rat DRG cells and a rat Schwann cell line (RSC96) for our experiment. We first administered ES alone to DRGs and selected different parameters for the study. To date, there have been many studies on ES, but there has been no relevant research on the optimal stimulation intensity. Moreover, different cells have different susceptibilities to ES, and the same treatment may exert different effects on different cells. For example, in observing the role of ES in wound healing, Wang Y selected different impulses under the 100 mV/mm electrical field as study parameters^35^, whereas Huang J chose 100 mV/mm, 300 mV/mm, 500 mV/mm and 1,000 mV/mm ES to treat Schwann cells^36^. Mccaig C D and Hotary K B selected parameters for ES that were within the physiological range of endogenous fields measured during embryogenesis or after injury^37,38^. Our experiments used electrical fields of 100 and 200 mV/mm and ES times of 0.5, 1, and 2 h. Our final experimental results showed that ES promoted the activity of DRGs. In addition, at 100 mV/mm and 200 mV/mm, cell viability decreased when the time was increased to 2 h compared to that at 1 h. Moreover, at an ES time of 1 h, the cell viability at 100 mV/mm was higher than that at 200 mV/mm. After a short period of ES, such as 0.5 or 1 h, the cells do not change significantly. However, excessive voltage or long-term stimulation may drastically alter the opening probability of certain ion channels on the surface of the DRG cell membrane or allow the cell membrane to repeatedly depolarize and repolarize, which destroys the normal physiological activity of DRG cells, thereby affecting cell growth, regeneration, and damage. Therefore, in the follow-up study, we used the following optimal ES parameters: 100 mV/mm for 1 h.

Many studies have shown that ES can cause changes in cell glutamate secretion, and this process is related to the treatment time^20,39,40^. During ES, cells are placed in an ES device, and it can be difficult to detect glutamate secretion. Therefore, we chose to culture cells in a new 6-well plate following ES to measure glutamate secretion. We observed that the amount of glutamate secreted by the DRG cells gradually increased after ES, and secretion was most rapid during the 6–8 h period. Therefore, we determined that the optimal co-culture time was 8 h for the subsequent experiment, when the glutamate secretion was highest, to observe the impact of glutamate on Schwann cells.

ES can promote the secretion of glutamate by DRGs, and studies have further shown that glutamate can promote the influx of calcium through binding to ionotropic glutamate receptors on oligodendrocytes, which in turn induces an increase in the release of oligodendrocyte exosomes^21^. In addition, one study showed that glutamate mediates Ca^2+^ signalling in oligodendrocytes^41^. Moreover, A Reddy found that increased intracellular calcium concentrations trigger the fusion of lysosomes and cell membranes, which induced exocytosis and increased the release of exosomes in a study of rat fibroblasts, myoblasts and the tubular epithelial cell membrane repair process^42^. Furthermore, Krämer-Albers E M revealed that the elevation of cytosolic Ca^2+^ levels stimulates exosome release^43^. Based on the above studies, we examined the changes in calcium concentrations in Schwann cells after glutamate treatment. We found that this change was positively correlated with exosome secretion. Therefore, we hypothesized that glutamate triggers Schwann cell-derived exosome release and conducted a follow-up experiment. The results from these experiments confirmed that glutamate affects Schwann cell exosome secretion. When the glutamate concentration was 100 μmol/L, exosome secretion was the highest.

Excess glutamate has a cytotoxic effect. Previous studies showed that glutamate-mediated toxicity can affect oligodendrocyte viability, and this process is dependent on the glutamate intensity and exposure duration^44,45^. However, Carsten Frühbeis observed no apparent damage to cultured oligodendrocytes after glutamate administration for 5 h. These authors also investigated the integrity of the plasma membrane by evaluating lactate dehydrogenase (LDH) release and propidium iodide exclusion and found no detrimental influence of glutamate during the exosome collection period to exclude interference of membrane fragments released from dying cells^21^. With reference to this article, we selected concentrations of 50, 100 and 200 μmol/L, and the time was fixed at 5 h for administering exogenous glutamate treatment to Schwann cells. We also performed experiments to confirm that within 5 h, the highest concentration of glutamate (200 μmol/L) had no effect on Schwann cell activity. Additionally, we cultured DRG cells with glutamine-treated Schwann cell supernatant and found that it could stimulate the activity of DRG cells. This finding showed that there was a substance in the supernatant of Schwann cells that could promote DRG cell activity.

Nerve cells and glial cells constitute the overall neural network. In this study, we administered nerve cell ES at the cellular level, which led to a series of changes. Gordon T confirmed that ES plays a role in the regeneration of peripheral nerves in peripheral nerve injury-related diseases^46,47^. In addition, ES accelerates axon outgrowth across the nerve injury site after both immediate and delayed nerve repair, and cAMP, neurotrophic factors, and androgens all participate in this process. Taking the *in vivo* environment into consideration, the changes in nerve cells impacted the surrounding Schwann cells. The ES-mediated release of neurotrophic factors from Schwann cells, nerve growth factor in particular, suggests a possible role of stimulated Schwann cells in accelerating axon outgrowth from the proximal nerve stump of injured neurons^48^. BDNF, a member of the neurotrophin family, plays a central role in neuronal proliferation, differentiation, and survival during development^49^. It has been previously reported that ES promotes BDNF expression in motor neurons, glutamatergic neurons and DRG cells^26,50,51^. In our experiment, suitable parameters for ES promoted the activity of DRG cells and increased the secretion of glutamate. This process might be related to the upregulation of BDNF and other neurotrophic factors induced by ES. The process by which ES promotes the secretion of BDNF is calcium dependent. Gordon T showed that ES promoted cAMP upregulation and increased the release of neurotrophic factors, such as BDNF, while neurotrophic factors accelerated the activation of cAMP^47^. Therefore, glutamate-promoted calcium concentrations in Schwann cells in our study might be associated with cAMP and BDNF secretion. These studies could be a new topic for future research.

ES can increase glutamate levels in DRG cells, whereas glutamate promotes the secretion of exosomes in Schwann cells. Thus, we hypothesized that increased glutamate from DRG cells after ES causes changes in Schwann cell intracellular calcium levels and Schwann cell-derived exosome secretion. In this way, the exosomes produced by Schwann cells may play a role in the activity of DRG cells. Therefore, we constructed a co-culture model of DRG cells and Schwann cells and observed changes in neuronal activity after co-culture. After ES, the activity of cells co-cultured with Schwann cells increased compared to that of DRG cells cultured alone. This result is consistent with experiments using the supernatant to culture nerve cells. In the co-culture model, we pretreated the Schwann cells with the exosome secretion inhibitor GW4869. We found no significant difference in the activity of co-cultured cells and non-co-cultured cells in the presence of the inhibitor. However, under the conditions of co-culture and ES, adding GW4869 significantly reduced cell activity and increased apoptosis, which suggested a potential role of Schwann cell-derived exosomes in this process.

Although it is a well-accepted and effective means of treating SUI, the underlying mechanism of ES remains unclear^52–54^; if uncovered, this mechanism could provide many new targets for the treatment of SUI. In the present study, it was confirmed at the cellular level that ES can promote the activity of nerve cells. Moreover, this effect was more pronounced in the case of co-culture with Schwann cells, which is consistent with the *in vivo* environment. In this process, Schwann cell-derived exosomes may play a very important role, providing a potential theoretical basis for further exploration of the role of exosomes in future studies.

This study has potential limitations. In addition to the role of exosomes, the regulation of glutamate and calcium also plays important roles in the effects of co-culture on DRG cells after ES. Studies have confirmed that trans-synaptic transfer of Wnt signalling is accomplished by the release of exosome-like vesicles and the transport of these vesicles to Frizzled 2 receptors^55^, while Wnt signalling can further induce the secretion of exosomes, which carry Wnt signalling pathway components to act on distant cells^56^. However, the specific regulatory mechanisms have not been studied in depth and need to be verified in subsequent experiments. We intend to continue probing these questions and to determine whether other elements are involved in the effect of ES on SUI in our future studies.

## Materials and Methods

### Cell resource

DRG cells (Rat dorsal root ganglion cells) were purchased from Zhen Shanghai and Shanghai Industrial Co., Ltd. (Shanghai, China; Item number HZ-C644) and maintained in Dulbecco’s modified Eagle’s medium (DMEM; Jenom, Hangzhou, China) containing 15% foetal bovine serum (FBS; Gibco-BRL, Invitrogen Life Technologies, CA, USA), 100 U/ml penicillin G and 100 μg/ml streptomycin (Jenom, Hangzhou, China). RSC96 cells(Rat Schwann cells) were purchased from BOSTER Biological Technology Co., Ltd (Wuhan, China; Item number CX0271). These cells were also cultured in DMEM containing 15% FBS, 100 U/ml penicillin G and 100 μg/ml streptomycin.

### Electrical stimulation model

DRG cells were digested with 0.25% trypsin + 0.02% EDTA (Sigma-Aldrich, St. Louis, MO, USA) when they grew to 90% confluence in cell culture flasks. Then, DMEM containing 15% FBS was added to the cell precipitate after centrifugation to obtain cell suspensions. The cell suspensions (2 ml) were then uniformly transferred onto the culture plate and placed in an incubator under 5% CO_2_ and 37 °C for 24 h. When the cells were adherent to the wall and full, the culture plate containing cells was transferred to a round petri dish (diameter: 18 cm) loaded with 80 ml of DMEM (containing 5% FBS, 100 U/ml penicillin G and 100 μg/ml streptomycin). Two round tubes (diameter: 1.5 cm) were fixed on both sides of the round petri dish, which was used to insert an agar salt bridge. The other end of the salt bridge was inserted into saturated potassium chloride solution. One end of the linked silver wire was inserted into saturated potassium chloride solution, the other end of the wire was connected with the positive and negative pole of the power supply. This device was able to give direct current electrical stimulation to cells on the plate in the circular petri dish. In this section, the stimulation parameters were: 100 mV/mm or 200 mV/mm of electrical strength and 0.5, 1, or 2 h of electrical time. DRG cells were divided into seven groups: the non-electrical stimulation group, the 100 mV/mm 0.5 h group, the 100 mV/mm 1 h group, the 100 mV/mm 2 h group, the 200 mV/mm 0.5 h group, the 200 mV/mm 1 h group and the 200 mV/mm 2 h group.

### Cell co-culture

Co-culture of DRG cells with RSC96 cells was performed using a 24-mm Transwell® with a sterile 0.4-µm pore polyester membrane insert (Corning Co., Ltd, NY, USA). Schwann cells were kept on the upper membrane 24 h in advance and allowed to grow to adherence, making sure the density of the cells was 90% the following day. After DRG cells were given electrical stimulation, digested with 0.25% trypsin + 0.02% EDTA and uniformly cultured to the bottom plate of the Transwell plate using 1.5 ml of cell suspension, we replaced the upper chamber with 1 ml of new medium. The number of cells cultured to the upper plate was counted and set at 7–8*10^5^/ml in each repeat experiment to ensure that the number of cells co-cultured in each experiment was the same. During co-culture, the exosome secretion inhibitor GW4869 (Selleck Biotechnology Co., Ltd., TX, USA) was added to the upper DMEM with 15% exo-FBS (Systembio Exosome-depleted FBS, system bioscience, SBI, CA, USA). In this section, DRG cells were divided into eight groups according to undergoing electrical stimulation or not; undergoing co-culture or not; and undergoing GW4869 or not.

### Cell proliferation analysis

Following electrical stimulation, cells were washed with phosphate-buffered saline (PBS; Jenom, Hangzhou, China) two to three times, wiping the edge of the plate. Cells were digested with 0.25% trypsin + 0.02% EDTA, and DMEM containing 15% FBS was added to the cell pellet following centrifugation (200 × *g* at room temperature for 8 min) to obtain the cell suspension, which was adjusted to 7–8*10^5^ cells/ml. The DRG cells were co-cultured with RSC96 cells for 8 h, digested, and then mixed with DMEM containing 15% FBS to obtain a cell suspension. When manufacturing the cell suspension, we made sure the concentration was 2*10^6^ cells/ml using cell counting instrument. The DRG cell suspension (100 μl/well) was pipetted into a 96-well plate and subsequently incubated in 5% CO_2_ at 37 °C for 1 h. Then, Cell Counting Kit-8 (CCK-8) solution (10 μl/well; Beyotime Institute of Biotechnology, Shanghai, China) was added to each well and incubated in 5% CO_2_ at 37 °C for 1 h. Finally, the optical density was measured at a wavelength of 450 nm using a microplate reader (Victor3, PekinElmer, USA).

### Cell senescence and Hoechst apoptosis staining

A fluorescent microscope was used to observe cell senescence and apoptosis. DRG cell senescence was assessed using the Senescence β-Galactosidase Staining kit (Beyotime Institute of Biotechnology, Shanghai, China), and apoptosis was determined using Hoechst 33258 staining. Clean coverslips were soaked in 70% ethanol for 5 min and dried on a sterile clean bench. They were then washed with cell culture medium and then placed in 6-well plates. After electrical stimulation, the DRG cells were evenly planted on the coverslips in 6-well plates and co-cultured with Schwann cells. If the operation did not require co-culture, culture plate-containing cells were washed with PBS and placed into a clean dish for the next treatment.

For cell senescence, β-galactosidase dye fixing solution (1 ml) was added to cells for 15 min at room temperature. Following this, cells were washed three times with PBS, and 1 ml of working fluid dye (10 μl of β-galactosidase staining solution A, 10 μl of β-galactosidase staining solution B, 930 μl of β-galactosidase staining solution C and 50 μl of X-Gal solution) was added. Cells were incubated at 37 °C overnight. Images were obtained using a light microscope (BX51, Olympus Corporation, Tokyo, Japan). For Hoechst apoptosis staining, 0.5 ml fixative was added, and the cells were incubated at 4 °C overnight. The fixative was removed the following day, and the DRG cells were washed three times with PBS. Then, 0.5 ml of Hoechst 33258 staining solution was added to the coverslips in the dark for 5 min. DRG cells were then washed twice with PBS, and a drop of anti-fluorescence quenching sealing liquid was added to the slide. A coverslip was then placed on the slide, and the cells were ensured to contact the anti-fluorescence quenching sealing liquid. Then, a fluorescent microscope (BX51, Olympus Corporation, Tokyo, Japan) was used to observe the occurrence of apoptosis. The DRG cells showed a normal blue nuclei under a fluorescence microscope if the cells were normal and healthy, whereas the nuclei of apoptotic cells were partially or completely stained with a colour that was somewhat white.

### Spectrophotometry for glutamate secretion detection

Following electrical stimulation, cells were washed with PBS and digested with 0.25% trypsin + 0.02% EDTA, and then DMEM containing 15% FBS was added to obtain the cell suspension, which was adjusted to 7–8*10^5^ cells/ml. The cell suspensions were cultured in 6-well plates, and the supernatants from 2 h, 4 h, 6 h, 8 h, 10 h, 12 h were collected to measure the content of glutamate in the supernatant (as there were a large number of cells that did not adhere in the 2 h group, this group was discarded). A glutamic acid detection kit (Nanjing Jiancheng Bioengineering Institute, Nanjing, China) was used to detect the concentration of glutamate in the supernatants, performed in accordance with the manufacturer’s instructions. An automatic biochemical analyser (Chemray 240, Rayto Science and Technology Co., Ltd, Shenzhen, China) was used for testing, and the instrument automatically generated sample concentrations. In this section, DRG cells undergoing electrical stimulation or not were divided into two groups, each group contains five groups (4, 6, 8, 10, 12 h).

### Exosome extraction, concentration detection and transmission electron microscopy observation

Exosomes were extracted using ExoQuick-TC^TM^ Exosome Isolation Reagent (EXOTC10A-1, system bioscience, SBI, USA.). Schwann cells were uniformly cultured in six-well plates using DMEM with 15% exo-FBS (Exosome-depleted FBS Media Supplement, EXO-FBS-50A-1, system bioscience, SBI, USA.), penicillin (100 U/ml) and streptomycin (100 μg/ml). When the cell density reached 80% confluence, different concentrations of glutamate (Sigma-Aldrich Inc, USA, Item number V900450, the glutamate solids were dissolved in PBS solution, and a small amount of hydrochloric acid was added to adjust the pH to promote dissolution) were applied to Schwann cells (0 μmol/L, 50 μmol/L, 100 μmol/L, and 200 μmol/L) for 5 h of culture^21^. The operation after treatment was completed according to manufacturer’s instructions. Briefly, biofluid was collected and centrifuged at 3,000 × *g* for 15 min to remove cells and cell debris. Then, the supernatant was transferred to a sterile vessel, and the appropriate volume of ExoQuick-TC was added to the bio-fluid followed by overnight refrigeration. The ExoQuick-TC/biofluid mixture was then centrifuged at 1,500 × *g* for 30 min. The supernatant was then aspirated, and the exosome pellet was resuspended in 100–500 µl using sterile 1 × PBS or specific buffer according to the downstream application.

Electron microscopic samples were prepared by the hanging drop method. A drop of sample suspension was dropped on a copper mesh (200 mesh) with carbon film using a small pipette. The copper mesh was allowed to stand for a few minutes after dripping. Then, excess liquid was removed from the edge of the copper mesh using filter paper, and the negative staining solution uranium acetate was dripped onto it for dyeing. Negative staining solution was then removed using filter paper. When samples were dry, they were observed using transmission electron microscopy (HT7700; HITACHI, Tokyo, Japan).

### Flow cytometric analysis: apoptosis and intracellular calcium concentration detection

Cell apoptotic rates were determined using Annexin V/PI (BestBio Science, Shanghai, China) double staining according to the manufacturer’s instructions. Briefly, cells from different groups were harvested, washed with ice-cold PBS twice and re-suspended in 400 μl of binding buffer. 5 μl of fluorescein isothiocyanate-conjugated Annexin V and 10 μl PI were added, followed by incubation for 20 min in the dark at room temperature. The apoptotic rate was analysed by flow cytometry using Flow Jo software 7.6 (BD Biosciences, NY, USA). Cells that stained positive for Annexin V and negative for PI were considered to be early apoptotic, whereas those that were positive for both were identified as late apoptotic cells. The apoptotic rates were expressed as the percentage of the total cell population.

The intracellular calcium concentration was measured using the Fluo-4 AM calcium ion fluorescent probe (Beyotime Institute of Biotechnology, Shanghai, China). After cells were treated with electrical stimulation or co-culture, they were digested with pure trypsin, and digestion was stopped using the original cell culture medium. Centrifuging at 1,000 × *g* for 5 min was performed to pellet the cells. Pre-cooled PBS (1 ml) was used to wash the cells twice. Then, 5 μM Fluo-4 AM (500 μl) was added, and cell samples were incubated at 5% CO_2_, 37 °C for 45 minutes, shaking gently several times. Samples without Fluo-4 AM served as a negative control. After incubation, centrifuging at 1,000 × *g* for 8 min was performed to precipitate cells, and washing with ice-cold PBS was performed twice to remove excess dye. Cells were resuspended with 500 μl of PBS and analysed using flow cytometry (BD LSR II; BD Biosciences, Franklin Lakes, NJ, USA).

### Statistical analysis

Statistical analyses were performed using SPSS 16.0 (SPSS, Inc., Chicago, IL, USA), and data are presented here as the mean ± standard deviation (SD). Groups were compared using analysis of variance (one-way ANOVA). Differences between two groups were determined using Student’s *t*-tests, and multiple means were compared by Tukey’s test. P < 0.05 was considered to indicate a statistically significant difference. Each experiment was repeated at least three times.

## Supplementary information

Supplementary Fig 1
