# Supplementary material for: Electrical stimulation enhances neuronal cell activity mediated by Schwann cell derived exosomes
Source: Sci Rep. 2019 Mar 12;9:4206. doi: 10.1038/s41598-019-41007-5 (PMC6414536; doi:10.1038/s41598-019-41007-5)
Supplement: Supplementary file 1 — Supplementary Fig 1 [file 41598_2019_41007_MOESM1_ESM.pdf]

supplementary information

Electrical stimulation enhances neuronal cell activity mediated by Schwann cell derived exosomes

Ming Hu[1], Li Hong [1*], Cheng Liu[1], Shasha Hong[1], Songming He[1], Min Zhou[1], Guotao Huang[1], Qian Chen[1]

[1]Dept. of Gynecology and Obstetrics, Renmin Hospital of Wuhan University, 238 Jiefang Road, Wuhan 430060, Hubei Province, China. Correspondence and requests for materials should be addressed to L.H. (email: lihong_whu@163.com)

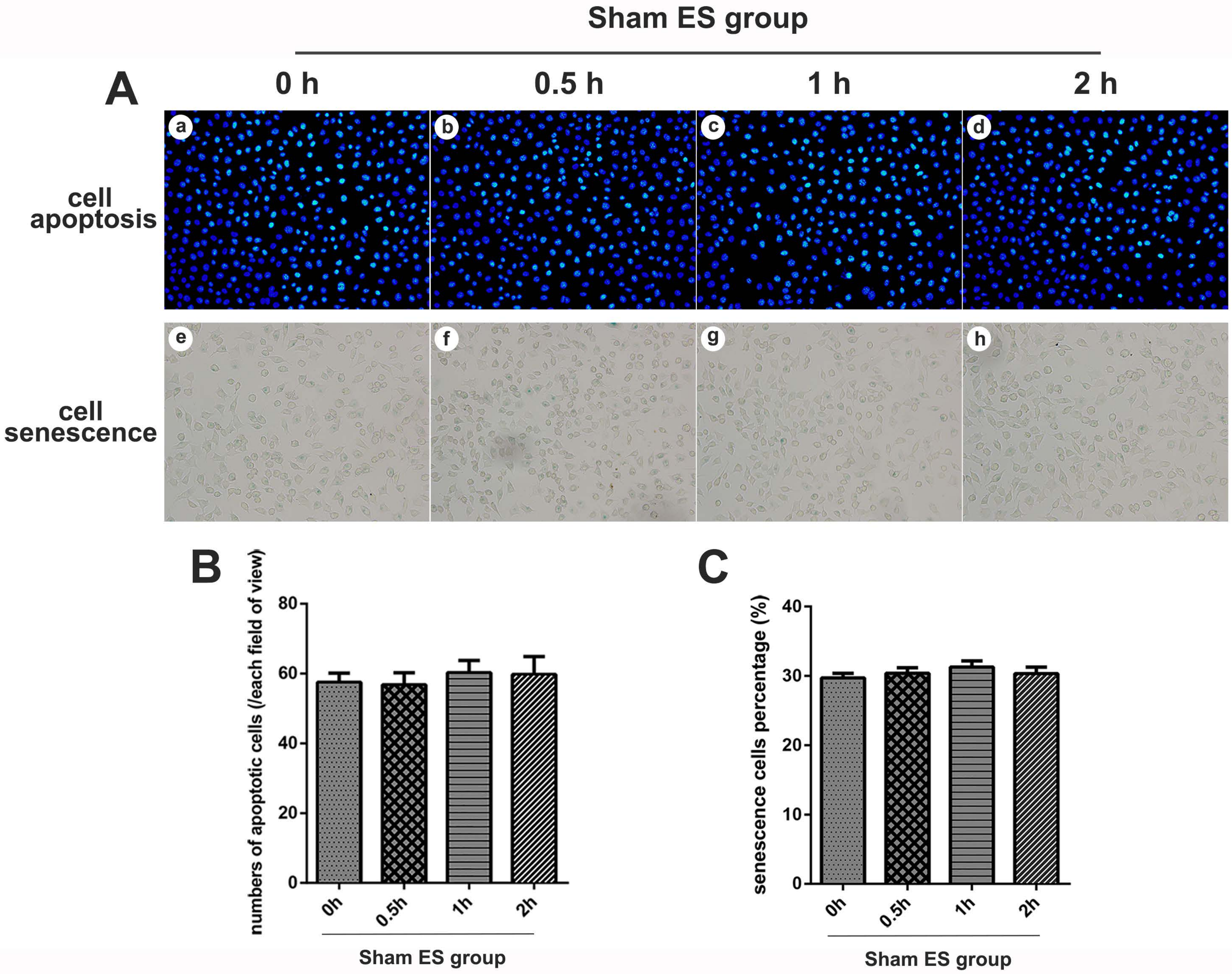

Supplementary Fig. 1 Apoptosis and cell senescence in the Sham ES group at each time point. (A a-d) DRG cell apoptosis staining at a 200× magnification. a, b, c, and d indicate the occurrence of apoptosis at 0 h, 0.5 h, 1 h, and 2 h, respectively. (A e-h) DRG cell senescence staining at a 200× magnification. e, f, g, and h indicate cell senescence at 0 h, 0.5 h, 1 h, and 2 h, respectively. (B) Quantitative analysis of apoptosis at each time point (0 h, 0.5 h, 1 h, and 2 h). (C) Quantitative analysis of cellular senescence at each time point (0 h, 0.5 h, 1 h, and 2 h).
